# Indigenous rights-based approaches to decolonising research methodologies in settler colonial contexts

**DOI:** 10.3389/frma.2025.1553208

**Published:** 2025-07-09

**Authors:** Peter Anderson, Zane M. Diamond, Thu Pham, Angela Baeza Peña, Carla Tapia, Levon Blue, Melanie Saward, Angelina Hurley, Kate Pecar, Amanda Kelly, Owen Forbes, Veronica Goerke, Jeane Diamond, Abby Cathcart, Chizu Sato, Koji Maeda, Russell Fox, Wren D. W. Howe

**Affiliations:** ^1^Indigenous Research Unit, Griffith University, Brisbane, QLD, Australia; ^2^Faculty of Education, Monash University, Melbourne, VIC, Australia; ^3^Carumba Institute, Queensland University of Technology, Brisbane, QLD, Australia; ^4^Office of the Deputy Vice-Chancellor (Indigenous Engagement), University of Queensland, Brisbane, QLD, Australia; ^5^School of Communication and Arts, University of Queensland, Brisbane, QLD, Australia; ^6^Creative Arts Research Institute, Griffith University, Brisbane, QLD, Australia; ^7^College of Nursing and Health Sciences, Flinders University, Adelaide, SA, Australia; ^8^College of Medicine and Dentistry, James Cook University, Townsville, QLD, Australia; ^9^Commonwealth Scientific and Industrial Research Organisation, Australian Capital Territory, Canberra, ACT, Australia; ^10^Equity and Inclusion, Student Life and Community, Office of the Deputy Vice-Chancellor, Academic and Indigenous Stewardship, Biodiversity and Environment Group (ISBE), Curtin University, Perth, WA, Australia; ^11^Success and Teaching Advancement (Learning and Teaching Unit), Queensland University of Technology, Brisbane, QLD, Australia; ^12^Division of Arts and Sciences, College of Liberal Arts, International Christian University, Tokyo, Japan; ^13^Faculty of Education and Integrated Arts and Sciences, Waseda University, Tokyo, Japan; ^14^Faculty of Education, School of Educational Psychology and Counselling, Monash University, Melbourne, VIC, Australia

**Keywords:** Indigenous rights-based research, decolonisation, research methodologies, education, settler colonialism, Indigenous knowledge, Indigenous people as data, protocols of engagement

## Abstract

**Introduction:**

Indigenous knowledge and perspectives continue to be misrepresented and misunderstood in settler colonial states, including within academic circles. This is particularly the case in the field of research, where non-Indigenous researchers continue to design and conduct research in their field of expertise without appropriate collaboration and guidance from Indigenous experts.

**Method:**

We explore the Indigenous rights-based approach (IRBA) as a means of decolonising research methodologies, focussing on the Australian context as a case study, where an Aboriginal Australian higher education expert has worked in a dyadic relationship with one Aboriginal and 16 non-Aboriginal subject experts to develop their knowledge, skills, and understanding of how to employ IBRA in their research. After working collaboratively, it became possible to analyse the similarities and differences in the use of IBRA across various fields of study.

**Results:**

Our analysis reveals five key aspects that were revealed during the implementation of the Indigenous rights-based approach: (1) Indigenous People as Data, (2) Protocols of engagement, (3) Privileging Indigenous Knowledge Systems, (4) Community Benefit, and (5) Tackling Doctoral Research Training.

**Discussion:**

We found that an Indigenous rights-based approach is crucial for decolonising research in settler colonial states such as Australia. Working in a dyadic partnership between an Indigenous higher education expert and academic researchers across several disciplines, we have seen an emergent approach to researching with Indigenous Peoples that allows non-Aboriginal researchers to work with Indigenous people in a manner that is ethical, relevant, and significant for Indigenous communities, contributing to place-based reconciliation and Indigenous community empowerment.

**Conclusion:**

We recommend how non-Indigenous researchers can collaborate with their universities to successfully implement an IRBA. Critically, this will require each university to employ Indigenous higher education experts who will lead and support professional development in research with non-Aboriginal people and communities. This will require a fundamental shift in how research is conceptualised, conducted, and disseminated.

## 1 Introduction

Australia's ongoing identity as a settler colonial state of the British Empire means that the path to decolonisation is littered with good intentions and obstacles, and any efforts to dismantle colonial frameworks must take place within colonial structures. The British system of governance, which was imposed upon Aboriginal and Torres Strait Islander peoples, lies at the heart of Australia's Constitution, driving cultural narratives and policies related to land rights, resource allocation, education, and research. This complexity is further compounded by the fact that most Australians are of British descent (Australian Bureau of Statistics, 2021) and inhabit lands colonised by their ancestors, reinforcing a self-perpetuating cycle resistant to change. Genuine decolonisation will necessitate dismantling the legacy of now-embedded colonial power structures. This paper demonstrates how an Indigenous rights-based approach (IRBA) can decolonise research methodologies.

Research, being inextricably entwined with the production and reproduction of theory, has proved vulnerable to pseudoscientific theories of the Enlightenment, which promoted notions of racial superiority in which European civilisations were seen as the pinnacle of human progress. At the same time, Indigenous peoples were understood to be primitive and uncivilised (Blyton, 2022). Theoretical concepts such as *terra nullius* and Social Darwinism were used to justify the colonisation of lands and the violation, subjugation, and genocide of Indigenous peoples. In Australia, Indigenous peoples were excluded as Australian citizens with sovereign rights. Early anthropological theory, for example, actively participated in this process using collected Indigenous ancestral remains to justify emerging evolutionary theories that regarded Aboriginal people as a “doomed race”, needing to be studied before inevitably dying out (McGregor, 1993).

Despite decades of calls for decolonising research practises since the 1970s, the Australian academic landscape continues to default to Western epistemological approaches that frequently misinterpret or marginalise Indigenous ways of knowing (Fredericks, 2009). In Australia, rather than recognising Indigenous connexions to Country, such colonially approved theories continue to view the land as a resource, influencing environmental and Indigenous land rights policies. Equally, Australia's higher education system is rooted in Western knowledge and colonial political and economic structures. Thus, universities and academic researchers received only superficial professional development about how to hear and appreciate Indigenous knowledge and voices, leading to ongoing socio-economic challenges for Indigenous peoples. In *Dhoombak Goobgoowana: A History of Indigenous Australia and The University of Melbourne*, Vice-Chancellor Maskell (2024) writes that the book challenges the “rosy … view of the University's past engagements with Indigenous people” (p. xi) by showing how it worked with colonial administrators to justify policies of dispossession, child removal and cultural destruction under the guise of scientific progress and civilisation.

Despite the growing awareness of Indigenous rights and knowledge systems and the need for decolonisation, research paradigms and methodologies remain deeply rooted in Western epistemologies and ontologies (Harrison and Sellwood, 2016; Higgins and Kim, 2018; Tuhiwai Smith et al., 2019), perpetuating systems of knowledge production that marginalise, misrepresent or entirely exclude Indigenous perspectives, experiences and ways of knowing (Chilisa, 2012; Getty, 2010; Tuhiwai Smith, 2021). The decolonisation of research methodologies is urgently needed to address the ongoing impacts of colonialism in academia. However, this involves more than a tokenistic inclusion of Indigenous peoples, views or topics; instead, it requires a fundamental shift in how research is conceptualised, conducted and disseminated. Research must also bring genuine benefits to Indigenous communities (Braun et al., 2013; Rigney, 1999). In other words, the aim is to conduct research *with*, not *on*, Indigenous peoples.

We argue that central to this shift is adopting an Indigenous rights-based approach (IBRA) to research, which is particularly relevant to colonial settler nations that have yet to decolonise their institutions and practises. This paper presents a case study focused on the Australian university context and does not seek to represent a universal Indigenous experience. By centring Indigenous rights from an Aboriginal Australian standpoint, this approach can transform research, policy and practise in ways that support Indigenous self-determination and social justice initiatives and contribute to global decolonisation efforts.

## 2 Global context of Indigeneity and Aboriginal Australian specificity

Indigenous research rights have been investigated in various nations that were once colonised. Research conducted in New Zealand (Wolfgramm et al., 2020) challenges deficit-based paradigms and emphasises Indigenous sovereignty by including Māori worldviews. Indigenous women's rights and roles in political and social activities are the subject of some research conducted in Canada (Gricius and Martel, 2024). In the meantime, environmental and land rights protection are key research areas in the United States (Lambert, 2014).

Similarly, studies conducted in Latin America (see Bonilla, 2019; Bose, 2017; Loncon, 2020; Zavala, 2020) examine Indigenous-led constitutional amendments that acknowledge self-governance, highlighting Indigenous dispossession, the land exploitation consequences, and water rights. Research has also been done on Indigenous women's leadership and the revival of Indigenous languages. Connell (2007) uses Southern Theory to explain how Latin American countries, particularly in terms of Indigenous peoples' colonial past, are comparable to Australia. Western paradigms have typically influenced knowledge production in these scenarios, usually portrayed as universal truths. As a result, decolonising research plays a crucial role in amplifying Indigenous voices (Baeza, 2022), and embracing these perspectives helps to inform and strengthen our Indigenous rights-based methodology.

The field of education offers particularly stark examples of how research has been weaponised against Indigenous communities. In the past, educational research provided a pseudoscientific rationale for assimilationist policies, most notably in the residential school systems of Canada and the United States, along with Australia's Mission Schools. The explicit mission of these schools was a systemic approach to cultural genocide that continues to reverberate through generations of Indigenous communities. (Anderson et al., 2024) show how colonial educational practises supported by contemporary research paradigms have created complex patterns of intergenerational trauma that continue to undermine Indigenous peoples' experiences of education. This trauma is manifested through individual educational outcomes and community relationships with academic institutions and research practises.

The history of implementing Indigenous decolonised research methods in Australia shows that international Indigenous research frameworks often fail long-term because they cannot fully encompass the complexity and nuances of Aboriginal Australian knowledge and practises. Most academic researchers in Australia are not Aboriginal, and their formal research training rarely covers Indigenous or specifically Aboriginal approaches in detail. Critical examination of research training in Australia in the Aboriginal domain is urgently needed because there is, as yet, little system-level agreement about what constitutes the skills and knowledge base for research by non-Indigenous researchers, either in the general sense or within their specific cognate area. The design and conduct of research commonly lack aspects that are highly important in Australian Aboriginal communities^1^, namely cultural safety, reciprocity, and meaningful community engagement—essential elements for ethical research with Indigenous communities.

While recent years have seen the emergence of frameworks such as Dadirri (deep listening) and Yarning (storytelling as a methodology) (Kennedy et al., 2022), their integration into mainstream research practises remains inconsistent and often superficial. Some non-Indigenous researchers attempt to implement such frameworks to guide their research, but frequently lack the foundational training required by their discipline to do so correctly. Unfortunately, most researchers shy away from doing anything for fear that they would do something incorrectly and unintentionally offend Aboriginal people, highlighting the ongoing need for structural transformation of research institutions and methodologies that centre Indigenous knowledge systems rather than merely accommodating them within dominant paradigms.

The equally pressing concern internationally of non-Indigenous researchers trying to use Indigenous research approaches is that this has led to a practise known as pan-Indigenisation, which generalises various Indigenous cultures and knowledge systems while obscuring significant distinctions between communities and contexts. Applying methodologies developed by Māori, Native American, or First Nations scholars to Australian Aboriginal and Torres Strait Islander settings without contextual adaptation can reinforce colonial knowledge extraction rather than promote genuine decolonisation (Skille, 2022).

This structural imbalance extends beyond research practises and into leadership within Australian higher education. Unrealistic expectations are often imposed on a small number of Indigenous leaders in universities, holding them solely accountable for all Indigenous outcomes within their institutions. As reported by the Minderoo Foundation Bankwest Curtin Economics Centre Murawin (2022), Indigenous employees are underrepresented mainly in senior management and executive leadership roles across various workplaces. To address this, we argue that it is critical to professionally develop non-Indigenous researchers with the knowledge, skills, and strategies needed to develop a sound understanding of Indigenous rights and their relevance in all aspects of research, both individually and as leaders in their respective disciplines.

## 3 Indigenous Rights–Based Approach (IRBA) to research methodology

The United Nations Declaration on the Rights of Indigenous Peoples (UNDRIP) (United Nations, 2007) affirms the rights of all Indigenous peoples to maintain their distinct institutions and participate in every aspect of society, including academia (Davis, 2016; United Nations, 2007). For decades, Indigenous scholars and activists have criticised Western research paradigms and argued for a radical transformation of research methodologies that centre Indigenous world views (see Kovach, 2015, 2021; Martin and Mirraboopa, 2003; Nakata, 2007; Nakata et al., 2014; Porsanger, 2004; Rigney, 1999, 2006; Tuhiwai Smith, 2021; Wilson, 2008). However, Coulthard (2014) and Simpson (2017) highlight the limitations of human rights-based frameworks in colonial states, arguing that Indigenous research sovereignty requires genuine decolonisation. Torres Strait Islander scholar Nakata (2007) highlights the complexities of researching at the “cultural interface” between Indigenous and Western knowledge systems, which can lead to a lack of action by Australian research institutions.

Recognising the complexities of the work, Australian scholars have provided a foundational understanding of an Indigenous rights–based approach and its potential to further the processes of decolonisation by centring Indigenous experts at the heart of academic research and teaching (Anderson et al., 2024; Anderson and Ma Rhea, 2018; Langton et al., 2009; Ma Rhea, 2015, 2014). This approach is based on the premise that Indigenous rights, values, and world views must be at the centre of any research involving Indigenous peoples. It acknowledges Indigenous peoples' sovereignty and right to self-determination, including the production and dissemination of knowledge, and is essential for decolonising research methodologies. This context-relevant IRBA also allows for the multicultural nature of Australian Aboriginal communities to be considered.

### 3.1 Developing a method of professional development in IRBA

Anderson, an Aboriginal academic leader and higher education expert, has worked with senior leaders and academics across multiple disciplines in several Australian universities to guide non-Indigenous academics in developing an Indigenous rights-based approach that enables meaningful engagement with Indigenous matters. Guided by the UNDRIP (United Nations, 2007), he has developed a collaborative methodology that has addressed and overcome the professional and institutional barriers faced by academics in the development and conduct of research.

Underpinning this work is the Senior Leadership Capability Model (SLCM) (Anderson et al., 2023) that advances Indigenous research methodologies by centring Indigenous leadership while engaging with Western academic structures. Rather than positioning Indigenous knowledge as an adjunct to dominant paradigms, the SLCM creates a replicable model for transforming disciplinary approaches, embedding Indigenous ways of knowing and being into institutional frameworks. A key aspect of the SLCM model is its emphasis on meaningful cross-cultural collaboration, establishing protocols that ensure partnerships between Indigenous and non-Indigenous scholars are built on reciprocity, respect, and shared authority. Furthermore, the SLCM demonstrates how Indigenous rights frameworks can be operationalised within specific disciplines, shifting from symbolic inclusion to genuine structural change. By strengthening leadership capacity among Indigenous and non-Indigenous university executives, the model balances responsibilities, addresses systemic inequities, and drives transformative change within Australian higher education. This approach strengthens Indigenous research methodologies and challenges the status quo, advocating for institutions to move beyond cultural awareness and embrace sustained, impactful decolonisation efforts.

This paper offers an insight into an approach successfully developed in dyadic relationships between Anderson and 17 non-Aboriginal academics. The academics who are also co-authors of this paper include Indigenous scholars from Canada and Chile, as well as non-Indigenous scholars from Australia, Vietnam, Japan, and Singapore, whose disciplines encompass the social sciences, natural sciences, health-related fields, and the arts. Anderson established and upheld Indigenous leadership throughout each dyadic professional development process within discrete research projects, creating an approach where Aboriginal expertise guided disciplinary adaptation rather than the reverse.

Over time, these dyadic collaborations evolved into an emergent, Indigenous rights-based approach that could be adapted for discipline-specific applications while retaining some identifiable common elements. To enable the analysis of these dyadic partnerships, Anderson synthesised data from the co-authors of this paper to develop an understanding of IRBA's strengths and limitations.

During the professional development phase of the work, each of these authors collaborated with and worked under Anderson's guidance to learn about and incorporate the Indigenous rights-based approach into their research and practise. Building on the foundation of Aboriginal leadership, this work adopted a dyadic collaboration process, where each non-Indigenous academic worked directly with Anderson to foster meaningful cross-disciplinary change. This collaborative method began by identifying the disciplinary barriers that hinder the implementation of Indigenous rights, acknowledging that many academic fields still operate within frameworks that marginalise Indigenous ways of knowing. Through ongoing dialogue and joint problem-solving, the collaborators developed discipline-specific applications of Indigenous rights principles, ensuring that these frameworks were treated not as abstract concepts but as practical tools for reshaping research practises. The process also involved systematically documenting the evolution of the IRBA from research design to implementation, capturing the steps taken to integrate Indigenous knowledge and values into disciplinary approaches. Finally, outcomes were evaluated within the context of each academic field, allowing for a nuanced understanding of how Indigenous rights can be and operationalised in contextually relevant and impactful ways. Each dyadic collaboration reinforced the role of Indigenous leadership and equipped non-Indigenous scholars with the insights and strategies needed to support sustained, transformative change.

As complex challenges require multiple perspectives (Brondizio and Tourneau, 2016; Whyte, 2018), a cross-disciplinary approach is methodologically necessary to demonstrate the applicability of Indigenous rights-based approaches across diverse knowledge domains. The benefit of this approach is that it builds bridges between knowledge systems, and practical implementation necessitates diverse expertise. Berkes (2009) documents successful knowledge co-production where scientific and Indigenous knowledge systems complement each other through collaboration across disciplines. Tuhiwai Smith et al. (2019) and Nakata et al. (2012) have also noted that while single-discipline approaches often reinforce colonial power dynamics by privileging Western epistemologies (Smith, 2012), cross-disciplinary methodologies create what Nakata et al. (2012) refer to as the cultural interface, where diverse knowledge systems engage meaningfully, producing a more comprehensive understanding and effective practical applications (Kimmerer, 2013).

The multiple authorship of this paper allows Anderson and his colleagues to reflect on the collaborative nature of knowledge production. Within Western journal conventions, it may seem unusual to position an Indigenous expert as authoritative, which could draw criticism. However, it is necessary to explain the approach developed by Anderson to provide insight into the effectiveness of the IRBA in the decolonisation of research. Without the leadership of an Aboriginal higher education expert, the approach cannot work. Anderson also invited the co-authors to contribute to this paper, acknowledging the necessity of including diverse disciplinary expertise and ensuring that the IRBA benefits from multiple perspectives. It also embodies his Aboriginal relational approach to knowledge creation, which emphasises shared responsibility, reciprocity, and collective wisdom. By bringing together scholars from diverse disciplines, he honours what Wilson (2008) terms “relational accountability” in Indigenous research paradigms, recognising that knowledge production is inherently collaborative and contextual.

## 4 Findings: transforming research to an Indigenous Rights-Based Approach

The journey towards decolonising research methodologies in settler colonial contexts such as Australia necessitates fundamentally reimagining how knowledge is produced, validated, and shared. Central to this transformation is adopting an Indigenous Rights-Based Approach (IRBA), which prioritises Indigenous sovereignty, self-determination, and community benefit. Through dyadic collaborations between an Aboriginal higher education expert (Anderson) and 17 non-Indigenous academics across diverse disciplines, this study explores the practical implementation of IRBA in the Australian context.

Our analysis reveals five critical aspects that emerged during this process:

- Indigenous People as Data—Challenging deficit narratives and centring Indigenous perspectives in data collection and analysis.
- Protocols of Engagement—Establishing ethical, reciprocal relationships with Indigenous communities.
- Privileging Indigenous Knowledge Systems—Validating and integrating Indigenous epistemologies into academic research.
- Community Benefit—Ensuring research outcomes directly serve Indigenous priorities and needs.
- Tackling Doctoral Research Training—Addressing systemic barriers for Indigenous scholars and redefining supervisory practises.

These aspects powerfully demonstrate how IRBA transforms research from an extractive practise into a vibrant force that empowers Indigenous communities, honours cultural protocols, and actively dismantles colonial frameworks. In the following sections, we will explore these dimensions in depth, offering rich insights from collaborative experiences while highlighting the significant challenges and abundant opportunities for decolonising research methodologies within settler colonial institutions.

### 4.1 Aspect 1: Indigenous people as data

#### 4.1.1 Case study 1: transforming statistical approaches through Indigenous rights

This case study examines how Anderson and Forbes' collaboration led to fundamental methodological shifts in quantitative research. This work illustrates how statistical analysis, traditionally a tool that reinforces deficit narratives about Indigenous peoples, can be reimagined through an Indigenous rights-based lens to create strengths-based analytical frameworks while maintaining methodological rigour.

Dyadic collaborations revealed that traditional research approaches have consistently positioned Indigenous peoples primarily as data sources instead of as knowledge holders or partners. Under Anderson's guidance, non-Indigenous researchers critically examined how their disciplinary methodologies reinforced deficit narratives, mainly through comparative analyses that measured Indigenous achievement against non-Indigenous benchmarks. This understanding prompted a fundamental methodological shift towards approaches centred on Indigenous perspectives and prioritising within-community analysis.

Forbes's collaboration with Anderson exemplifies this transformation, resulting in rights-based statistical methodologies that resist deficit narratives while maintaining analytical rigour. The development of the within-cohort peer-matching approach represents a concrete outcome that operationalises Indigenous rights principles within quantitative research frameworks, directly challenging how seemingly objective statistical methods have historically reinforced colonial perceptions. Forbes explains how the Indigenous rights-based approach has transformed his research practise:

Anderson's mentorship and collaboration early in my research career have been transformative in shaping my approach to Indigenous rights–based research and statistical analysis. His guidance has been instrumental in co-developing frameworks that centre Indigenous data sovereignty and embed UNDRIP principles directly into foundational statistical methodologies. This influence is particularly evident in the development of rights-based approaches for within-cohort Indigenous peer-matching analyses, which resist deficit-oriented narratives while advancing methodologically rigorous, culturally informed statistical techniques to understand patterns of educational performance among Indigenous students. (November 2024)

In the Australian context, leading Indigenous data expert Walter (2018) describes an “Indigenous data paradox” in which data about Indigenous peoples overwhelmingly focuses on “5D Data”: Difference, Disparity, Disadvantage, Dysfunction, and Deprivation. This deficit-based paradigm portrays Indigenous peoples as problems to be fixed rather than communities with distinct strengths and priorities. Walter argues that Indigenous data sovereignty, wherein Indigenous peoples control the collection, analysis, and use of data about themselves, is essential for shifting away from these harmful narratives. This perspective aligns directly with Forbes and Anderson's emphasis on within-community analysis over deficit-based comparisons.

Data collected about Indigenous Australians since the mapping of Australia by Dutch, English and French explorers in the late 1700s and the scientific expeditions led by Charles Darwin in 1831–1836, have been used to justify policies and practises related to Indigenous affairs. These data have generated the perception that the intelligence and academic capacity of Indigenous peoples are somehow deficient, resulting in contemporary government programs aimed at “closing the gap” between Indigenous and non-Indigenous students in terms of academic achievement.

In 2009, the Australian Government began standardising student assessments through NAPLAN, making it possible to analyse and compare government data on Indigenous student achievement. This proved to be significant because, as Langton et al. (2009) argued, a comparable dataset across all states and territories was crucial to understanding the reality of Indigenous student achievement. Anderson et al. (2024) answered this call by developing a within-cohort peer-matching methodology to analyse NAPLAN data from 2009 to 2019. However, the *a priori* assumptions underpinning the traditional statistical approach could not be demonstrated without undertaking a re-analysis of the primary sources. By exclusively comparing Indigenous students with each other, the new methodology shifted the focus away from historical deficit-based narratives and towards examining patterns of success within Indigenous communities thereby being able to hypothesise contextual factors that influence educational outcomes. This approach fully embraces and respects Indigenous data sovereignty by privileging Indigenous perspectives, experiences and definitions of success rather than focusing on the achievement “gap” between Indigenous and non-Indigenous students. The approach contributes to the broader goal of decolonising educational research and policy by privileging Indigenous perspectives.

This methodological innovation developed through the dyadic partnership between Anderson and Forbes uncovered that Indigenous students demonstrated remarkably consistent high achievement in specific contexts, challenging prevailing narratives about an “achievement gap”. Education departments in three Australian states have since adopted elements of this approach to redesign their assessment frameworks, demonstrating how an Indigenous rights-based methodology can directly influence policy development while maintaining rigorous statistical standards.

### 4.2 Aspect 2: protocols of engagement

#### 4.2.1 Case study 2: redefining research engagement through Indigenous rights

This case study documents Anderson's collaboration with Baeza Peña to develop engagement protocols that genuinely honour Indigenous community decision-making processes. Their partnership reveals how conventional research timelines and consent procedures systematically undermine meaningful Indigenous participation and demonstrates how alternative approaches can transform research relationships. Research consent forms cannot conceal the truth: most “engagement” with Indigenous communities remains transactional, not transformational. With Aboriginal guidance, the research design exposed how institutional timelines and academic jargon render free, prior, and informed consent meaningless. Baeza Peña's work unveils the radical alternative—protocols based on sustained relationship-building, not rushed ethics approvals. Her model proves that authentic engagement requires dismantling academia's extractive clock and honouring Indigenous decision-making processes that unfold across seasons, not semesters. Her research was able to demonstrate that data collection isn't about checking boxes; it's about breaking chains. She found that when agreed research timelines aligned with community rhythms—not grant cycles—she was not simply following cultural protocols; she was restoring research sovereignty to her community.

Protocols of engagement that ensure full, free, and informed consent are essential for developing effective research partnerships with Indigenous peoples and communities. However, establishing these protocols is challenging, especially when traditional research frameworks intersect with complex community structures and identities (Brunger and Wall, 2016; Saward, 2024a,b). Engaging Indigenous communities in research relies on trust-building and in-depth conversations about the potential benefits of the research for the community (Baeza Peña et al., 2023). Indigenous communities have multiple layers of authority and complex decision-making processes that may not be readily apparent to external researchers. Therefore, consent must be considered throughout the research lifecycle, requiring sustained engagement and relationship-building (Smylie et al., 2014).

According to Baeza Peña, learning about the Indigenous rights-based approach has:

… highlighted the necessity of acknowledging and respecting Indigenous communities' rights, perspectives and needs in educational research and practise. Using this approach, I was able to turn my research findings into a holistic model that emphasises the interconnectivity of the Indigenous community, the school context, outside help and personal resources … and promotes a commitment to the values of equality and justice … [ensuring] that my research consistently prioritises the benefits and wellbeing of Indigenous children. (November 2024)

Baeza Peña's (2023) research in rural Chilean Indigenous communities demonstrates how transforming engagement protocols produces fundamentally different research outcomes. When university ethics timelines demanded quick community consultations, she instead implemented a seasonal engagement model where research activities aligned with community rhythms rather than academic calendars. This approach extended the project timeline by 8 months but resulted in community elders formally endorsing the research and actively contributing to the design of the methodology. The resulting data revealed critical insights about educational needs that would have remained hidden under conventional consultation models. This project established a cross-institutional protocol for Australian universities to guide engagement with Indigenous communities, featuring flexible timelines and explicit provisions for community ownership of research outputs.

Furthermore, Georke (2019) and Goerke and Anderson (2021) provide valuable insights into establishing ethical research relationships in universities. This highlights the importance of creating “right relationships” through deep listening and genuine partnerships rather than tokenistic consultations and emphasises that effective protocols must position Indigenous communities as active decision-makers rather than passive research subjects. By centring Indigenous rights, researchers can transform reconciliation from symbolic gestures into meaningful partnerships that respect Indigenous sovereignty and self-determination instead of treating engagement merely as a corporate social responsibility exercise.

Through dyadic partnerships with Anderson, researchers from various disciplines demonstrate the broad applicability of ethical engagement protocols. Baeza Peña's seasonal approach in Chilean communities, Saward's relational literary research, Goerke's institutional frameworks, and Hurley's community-centred methodology collectively illustrate how these principles transcend disciplinary boundaries. These diverse applications, spanning education, creative arts, governance, and community development, share a crucial insight: when Indigenous decision-making processes take precedence over institutional timelines, research yields more authentic knowledge that better serves community interests, representing a fundamental shift in how knowledge is co-created

### 4.3 Aspect 3: privileging Indigenous knowledge systems

#### 4.3.1 Case study 3: centring Indigenous epistemologies in academic research

This case study examines how Anderson's collaborations with authors Pham, Maeda, Yip and J. Diamond challenged institutional paradigms that systematically exclude Indigenous knowledge systems. Their work illustrates how research frameworks can be restructured to prioritise Indigenous epistemologies as sovereign knowledge systems instead of supplementary perspectives within Western academic traditions.

The collaborations revealed a fundamental contradiction: Western research paradigms routinely exclude Indigenous epistemologies, coercing Indigenous scholars into colonial frameworks regardless of the research's geographical location, whether Japan, Vietnam, Singapore, or Australia. With Anderson's leadership, non-Aboriginal researchers confronted how institutional power, embedded in funding systems, validation metrics, and academic hierarchies, actively suppresses Indigenous ways of knowing. This effort was not about superficial inclusion; it required dismantling the epistemological foundations of academia itself.

The result? A seismic redefinition of rigour where Indigenous knowledge systems are not merely included but recognised as sovereign, reshaping research itself. According to Pham,

the Indigenous rights–based approach has profoundly shaped my work by embedding principles of equity, self-determination and cultural integrity at the core of my research … [enhancing] my ability to support Indigenous student success while centring their voices and rights. (November 2024)

The privileging of Indigenous knowledge systems also applies to other countries. Maeda reflected that Anderson's research on the Indigenous rights-based approach has provided him with

… significant insight into the reconstruction of a higher education system in which Indigenous people play a central role as one way of restoring Indigenous sovereignty. One outcome of this is the planning and implementation of an Indigenous-led teacher training program at Monash University. This has also had a significant influence on my book, *Autonomy Development and Higher Education of the Australian Indigenous Community*. (November 2024)

Anderson P. J. et al. (2022b) conducted a meta-synthesis of studies on the issues and challenges faced by universities in terms of enhancing Indigenous student success. They found that universities generally rely on ameliorative approaches to address the assumed Indigenous deficit rather than being geared towards Indigenous success. In contrast, an Indigenous rights-based approach problematises the readiness of universities rather than the readiness of Indigenous students.

Dr Sun Yee Yip explains that using an Indigenous rights-based approach has significantly influenced her research and teaching practise:

In research, it serves as a frequent reminder to shift away from deficit thinking or needs-based perspectives and harness the strengths of my participants. In teaching student teachers, I made it a point to recognise the knowledge and perspectives of Indigenous people in everyday teaching and learning and to role model what it means for myself and my student teachers. (November 2024)

Based on their lecturing experiences in an initial teacher education program at Monash University, Anderson et al. (2018) employed an Indigenous rights-based approach to preserve Indigenous histories, languages, and cultures. Jeane Diamond commented,

The breadth and depth of information on Indigenous rights … enabled me to clarify for my students why the multicultural inclusivity approach continues to colonise Indigenous peoples in Australia … and that while Aboriginal people (and Torres Strait Islanders) have human rights along with migrants and refugees, they also have sui generis inalienable rights as the first peoples of this land. (November 2024)

This cross-disciplinary applicability reveals how privileging Indigenous knowledge systems is not confined to specific academic domains but has transformative potential across diverse fields. Researchers create more relevant, ethical, and effective institutional practises regardless of discipline by centring Indigenous epistemologies as sovereign knowledge systems rather than merely supplementary perspectives. This challenges the false universality of Western knowledge frameworks while opening new pathways for addressing contemporary challenges.

Integrating and prioritising Indigenous knowledge systems in research will lead to a fundamental shift in how knowledge is conceptualised, validated, and transmitted. However, this transformation will necessitate more than a superficial acknowledgement of Indigenous perspectives or simple methodological changes; it demands a profound understanding of Indigenous epistemologies and distinct ways of knowing, being, and doing (Wilson, 2008). Furthermore, the institutional barriers to Indigenous participation and leadership in research must be dismantled, including rigid structures as well as implicit funding and publication biases (Anderson et al., 2023; Anderson and Diamond, 2021; Cull et al., 2018). Significant investments in capacity building and resource allocation are required to cultivate Indigenous research leaders (McGregor et al., 2018).

Through the work of these authors, their dyadic partnerships with Anderson demonstrate that research academics in Australian, Vietnamese, and Japanese universities can adopt this IRBA in their Indigenous student support, alternative education, teacher preparation, and STEM education programs, illustrating how centring Indigenous knowledge systems creates more relevant and effective institutional practises.

### 4.4 Aspect 4: community benefit

#### 4.4.1 Case study 4: ensuring research serves Indigenous communities

This case study examines Anderson's work with Kelly and Pecar to reorient research objectives towards Indigenous community priorities. Their collaboration demonstrates how the integration of UNDRIP principles transforms research from an extractive academic exercise into a process that actively supports Indigenous self-determination and sovereignty. Kelly's curriculum redesign and Dr. Pecar's clinical optometry work each demonstrate a shift away from having Indigenous people exist within an extractive relationship as consumers of public health services rather than active participants in their wellbeing.

Kelly reflects on how the Indigenous rights-based approach:

… has significantly shaped my research by providing a framework to privilege the voices of Indigenous students, families and communities. By implementing principles within UNDRIP, this approach has influenced my work in curriculum development and Indigenous research by placing an emphasis on respecting Indigenous self-determination and embedding culturally appropriate pedagogies which prioritise Indigenous perspectives and knowledges. This approach has also guided my work in addressing systemic barriers and advancing educational practises that uphold the rights and aspirations of Indigenous students on their own terms. (November 2024)

Similarly, Pecar explains how:

… the rights-based approach, particularly through the lens of UNDRIP, has profoundly shaped my teaching, research and clinical practise in optometry. As a non-Indigenous ally, it is especially important to me that the principle of self-determination is both respected and prioritised across all aspects of my work, ensuring that Indigenous peoples have the autonomy to make decisions about their own lives, communities and health. (November 2024)

Blue's (2016) and Blue and Pinto's (2023) work on financial literacy highlights a commitment to community-defined benefits by collaborating with Indigenous communities and shifting from Western economic concepts to co-creating financial education frameworks that honour Indigenous practises and collective decision-making. It demonstrates Indigenous economic sovereignty and ensures outcomes that foster immediate financial wellbeing and long-term self-determination. This approach challenges financial literacy education by transforming it from an assimilationist practise into one that reinforces cultural values while addressing current economic needs.

Their projects, rooted in UNDRIP principles, transformed research from an academic exercise into a tool for community self-determination. Their learning from collaborations with Indigenous peoples and communities is explicit: impact must be measured by Indigenous priorities, not publication counts. Understanding that when research serves the communities it studies, it doesn't just generate knowledge; it generates justice.

The priorities of researchers and Indigenous communities regarding research design and knowledge dissemination often conflict (Anderson et al., 2024; Ball and Janyst, 2008). Conventional evaluation frameworks typically fail to capture the impact of research on Indigenous communities, necessitating new approaches that align with Indigenous values and priorities (LaFrance and Nichols, 2010). The deep-seated tensions between institutional and Indigenous protocols (Castellano, 2004; Weber-Pillwax, 2001) underscore the need for a fundamental shift in how research is conceptualised, funded, implemented, and evaluated (Kirkness and Barnhardt, 2001), ensuring that research provides genuine benefits to Indigenous communities. These principles must be situated within the broader movements of Indigenous resurgence and decolonisation to avoid superficial or romanticised interpretations of Indigeneity that frequently permeate Western academic discourse (Corntassel, 2012; Simpson, 2017).

Contemporary Indigenous scholarship increasingly emphasises methodological approaches that centre on Indigenous ways of knowing and being, including land-based methodologies (Wildcat et al., 2014) and ceremonial practise research (Wilson and Hughes, 2019). Similarly, integrating Indigenous languages into research methodology (McCarty and Nicholas, 2014) extends beyond mere translation purposes to encompass fundamental knowledge production and dissemination. These approaches transcend rights-based frameworks to engage with Indigenous epistemologies that predate colonial research paradigms.

Kelly's and Pecar's curriculum development projects highlight the transformative impact of prioritising community benefit shaped by Indigenous rights. Conceived initially as a standard curriculum review, the project was restructured under Anderson's guidance to ensure that Indigenous community priorities drove the research design. Instead of extracting community knowledge to inform university-determined objectives, the methodology reversed this relationship, establishing community councils that defined research priorities, methodology, and success measures. The resulting curriculum reflected Indigenous pedagogical approaches rather than simply adding Indigenous content to Western frameworks.

The collaborative efforts of these researchers demonstrate that ensuring community benefit transcends disciplinary boundaries. Kelly's curriculum development, Pecar's clinical practise, and Blue's financial literacy programs collectively illustrate how academics across diverse fields from healthcare to education to economic growth can successfully reorient research priorities to serve Indigenous communities. Their experiences reveal that when Indigenous-defined metrics, rather than institutional standards, measure research outcomes, the work produces more meaningful knowledge and actively supports Indigenous self-determination and sovereignty across multiple contexts.

### 4.5 Aspect 5: tackling doctoral research training

#### 4.5.1 Case study 5: decolonising doctoral supervision and research training

This case study documents Anderson's collaborative research with Blue, and Cathcart and Hurley's experiences with Anderson to restructure doctoral research training (Anderson P. et al., 2022). Their work reveals how conventional PhD paradigms systematically exclude Indigenous ways of knowing and demonstrates how an Indigenous rights-based approach to supervision can create pathways for Indigenous research methodologies to transform academic disciplines from within.

Cathcart's work reframes supervision through UNDRIP's decolonial perspective. Hurley's journey as an Aboriginal PhD student and now postdoctoral fellow reveals that an Indigenous rights-based approach provides the *raison d'être* to shape rigorous academic research methods that reveal the systems that seek to silence them. The limited research on Australian Indigenous doctoral research training focuses primarily on how universities can create culturally inclusive and “safe” spaces for Indigenous scholars while neglecting education's technical and scholarly aspects. High-quality supervision and training of non-Indigenous supervisors are crucial for the success of Indigenous doctoral candidates (Anderson and Hudson, 2020; Anderson et al., 2024, 2021; Anderson P. J. et al., 2022b; Nakata, 2007).

Cathcart commented that the Indigenous rights-based approach has:

… helped shape my thinking and practise in relation to … my work in research supervision and curriculum development, and recognising the legacy of colonisation in academic practise. In co-developing the Indigenous Perspectives and Knowledges in Learning and Teaching program at QUT, we consciously rejected approaches used elsewhere in [higher education] in framing our work not through a social justice, equity or cultural competency lens, but instead drawing on UNDRIP to challenge non-Indigenous academics to critically reflect on educational practise and knowledge systems that continue to marginalise the rights of Aboriginal and Torres Strait people. (November 2024)

Indigenous doctoral students, poised to become leaders of change in their communities, can merge their lived experiences with academic knowledge to challenge Western institutions and disciplines that have historically silenced Aboriginal voices (Anderson et al., 2021; Anderson P. J. et al., 2022b; Pham et al., 2024). Indigenous rights-based approaches may assist students and novice researchers in advancing decolonisation within their fields. Hurley emphasised the importance of an Indigenous rights-based approach that “centres sovereignty and prioritises a Blak voice … [Anderson's] focus on accrediting Aboriginal knowledge as essential tools for decolonizing Western systems has been invaluable in supporting my perspective as both an academic and a creative writer” (November 2024).

The collaborative work of these researchers demonstrates that transforming doctoral research training transcends institutional contexts. Cathcart's supervision frameworks, Hurley's creative research pathway, and the mentoring approaches of Anderson with Blue, Pham, and Saward collectively illustrate how academics across diverse fields, from institutional leadership to creative arts to student support, can successfully reimagine HDR education. Their experiences reveal that when doctoral training explicitly validates Indigenous knowledge systems and creates space for Indigenous sovereignty, it improves completion rates and actively contributes to the decolonization of academic institutions across multiple contexts.

## 5 An Indigenous rights–based approach: what have we learned?

In this paper, we identify five aspects related to the Indigenous rights-based approach: Indigenous people as data, protocols of engagement, privileging Indigenous knowledge systems, community benefit, and tackling doctoral research training. Despite the increasing acknowledgement of the need for fundamental change, academic and research institutions in settler colonial states continue to operate within colonial power structures, from the systemic underrepresentation of Indigenous scholars to the marginalisation of Indigenous knowledge systems. Anderson et al. (2023) call for an “Indigenous rights-based organisational consciousness” (p. 445) within institutions to address these structural inequities. This requirement extends beyond superficial reforms and rhetoric about reconciliation; it demands a fundamental transformation of institutional practises, policies, and cultural frameworks. This presents challenges and opportunities on the journey towards decolonisation. Successfully implementing Indigenous rights-based approaches will require attention to multiple domains of academia to challenge colonial power structures, including individual research practises, institutional policies, resource allocation, and cultural transformation. Employing an Indigenous rights-based approach has enabled us to navigate Nakata's (2007) “cultural interface” in ways that are acceptable not only to the authors of this paper but also to the Indigenous peoples and communities with whom they work, proposing what success may look like for Australia as it moves towards a decolonised future. This work fundamentally challenges the Indigenous/Western research binary by demonstrating how IRBA creates a transformative “third space” (Nakata, 2007) that respects sovereignty while engaging with institutional systems. As evidenced above, combining Indigenous priorities with disciplinary rigour moves beyond oppositional frameworks to relational accountability (Wilson, 2008).

### 5.1 Ensuring data sovereignty: overcoming the use of Indigenous people as data

Indigenous data sovereignty is based on the following key principles:

- Indigenous people having full control over the collection, ownership and application of information about them and their communities and territories.
- A rejection of deficit-based comparative methodologies.
- The centring of Indigenous perspectives, experiences and definitions of success in data collection and analysis.
- The prioritisation of within-community analysis over comparative frameworks.
- The alignment of research methodologies with Indigenous principles of sovereignty.
- The prioritisation of the interests of Indigenous communities over those of institutions.

We have learned that successful implementation of Indigenous data sovereignty will require:

- a fundamental transformation of institutional ethics approval processes,
- a willingness to challenge established data collection and analysis practises,
- new frameworks that respect Indigenous sovereignty and ways of knowing,
- methodological choices that actively challenge colonial power structures,
- the development of culturally appropriate analytical methods,
- the development of policies that align with Indigenous perspectives and priorities,
- the maintenance of an analytical focus within Indigenous communities.

Anderson et al. (2024) present a within-cohort peer-matching methodology that models how Indigenous data sovereignty can be successfully achieved. Instead of comparing Indigenous and non-Indigenous populations regarding academic achievement, the methodology focuses solely on patterns within Indigenous communities. It illustrates how methodological innovation can aid the decolonisation of educational research and policies while honouring Indigenous ways of knowing and measuring success. This approach can be conceptualised as “decolonisation” through transformation, utilising colonial methods to conduct strengths-based assessments, ultimately ensuring self-determination. Furthermore, by decolonising current research methods, both subtle and overt racism associated with the “acceptable” research space for Indigenous scholars may be addressed. The gatekeeping of existing research methods perpetuates the cascading negative effects of colonial structures within academia and government. An Indigenous rights-based approach can challenge these structures and promote self-determination throughout all facets of the research process.

### 5.2 Protocols of engagement: establishing clear and enforceable protocols

We have learned that incorporating Indigenous languages and cultural practises into academic institutions is crucial for transforming research. This must move beyond a tokenistic acknowledgement to a meaningful integration of Indigenous cultural practises. The development of institutional research policies that respect Indigenous rights and protocols will require:

- clear guidelines for engaging with Indigenous communities,
- oversight mechanisms that ensure compliance with Indigenous protocols,
- consultation processes that centre Indigenous voices in decision-making,
- accountability measures that ensure institutional responses to Indigenous concerns,
- resource allocation frameworks that support Indigenous research priorities through:

° careful attention to protocols governing language use,
° recognition of the sacred nature of linguistic knowledge,
° understanding the relationship between language and knowledge transmission,
° respect for community authority over linguistic and cultural resources.

Acknowledging and respecting diversity requires a modification of research methods to align with local Indigenous protocols. This requires researchers to:

- develop a deep understanding of specific community protocols,
- build meaningful relationships with knowledge holders,
- adjust timeframes and processes to accommodate local cultural practises,
- recognise the validity of diverse ways of knowing and knowledge transmission.

Baeza Peña et al. (2023) outline essential protocols for engaging with Indigenous communities and emphasise the importance of building authentic trust. Establishing this trust takes time and a commitment to relationship building, which includes engaging in meaningful discussions about the potential benefits of the research. Clear, jargon-free communication is crucial for informed consent. Research findings should amplify community voices rather than merely reflect the researcher's perspective and should be made available to Indigenous communities to enhance their lives.

### 5.3 Privileging Indigenous knowledge systems

Indigenous knowledge systems should be integrated into graduate curricula to foster epistemological plurality. This will necessitate re-evaluating the value of diverse types of knowledge and how this knowledge is transmitted, evaluated, and applied. This transformation will challenge conventional academic hierarchies while making room for Indigenous ways of knowing and being within institutional frameworks.

We have learned that overturning deficit-based narratives about Indigenous learners and communities will require a transformation of both research practise and institutional infrastructure by:

- developing research frameworks that privilege Indigenous perspectives and priorities,
- creating institutional policies that support Indigenous research methodologies,
- formulating evaluation criteria that recognise and value Indigenous ways of knowing,
- establishing support structures that facilitate Indigenous-led research initiatives,
- transforming funding mechanisms to support Indigenous research priorities,
- developing ethical guidelines that centre Indigenous rights and protocols.

Research organisations must genuinely embrace Indigenous approaches and knowledge systems (Anderson et al., 2023). This can be achieved by recognising that Indigenous leadership is typically communal, rooted in cultural knowledge and community ties, and based on consensus rather than individual authority. Organisations should not merely conscript Indigenous leaders into existing Western leadership structures; rather, they must cultivate environments that support Indigenous philosophies, collective decision-making, and community connexions. This transformation is essential to incorporate Indigenous perspectives and foster inclusive research practises.

### 5.4 Ensuring community benefit

Community benefit means that research directly serves the interests of Indigenous communities rather than merely fulfilling academic or institutional goals. Successfully achieving this depends on the following:

- Indigenous communities rather than researchers or funding bodies must determine research aims, priorities and measures of success based on their knowledge systems.
- The findings must be made accessible to Indigenous communities in culturally appropriate formats.
- The research process must strengthen Indigenous self-determination and sovereignty rather than perpetuating dependent relationships.
- Projects must build long-term capacity within communities through skills development, training and resource allocation.
- The benefits must be holistic, moving beyond theoretical academic outputs to include cultural revitalisation, community empowerment and practical improvements to the lives of Indigenous peoples.

We have learned that this will require structural changes in how research is:

- evaluated (i.e. in a way that supports Indigenous measures of value),
- funded (i.e. in a way that enables sustained community engagement and relationship building),
- conducted (i.e. in a way that meets Indigenous protocols and timeframes rather than rigid institutional schedules),
- disseminated (i.e. in a way that gives communities control over how their knowledge is shared and used).

A community benefit must be measured according to Indigenous metrics of success. Moreover, it should not be viewed as a one-off achievement but demands an ongoing commitment to reciprocal relationships, cultural protocols and Indigenous sovereignty over research processes. True success means transforming research from an extractive practise into one that actively supports Indigenous aspirations and wellbeing while challenging the colonial power structures within academia. This will require profound institutional change and genuine partnerships with Indigenous communities.

### 5.5 Rethinking doctoral research training

The recruitment and retention of Indigenous academics is another critical pathway to institutional transformation. This must extend beyond mere demographic targets to address the systemic barriers to academic participation faced by Indigenous peoples. Success in this area requires attention to multiple factors, including the development of:

- supportive academic environments that value Indigenous knowledge systems,
- mentorship and professional development opportunities specifically designed for Indigenous scholars,
- policies that recognise and support Indigenous scholars' community obligations,
- institutional support structures that acknowledge and address the unique challenges faced by Indigenous academics.

These efforts must be understood within the broader decolonisation and Indigenous resurgence contexts. Success will require a long-term commitment to change, including removing systemic barriers and developing institutional frameworks that serve Indigenous interests and priorities.

We have learned that respecting diversity means avoiding pan-Indigenous approaches, which risk perpetuating colonial practises of homogenisation and erasure, despite being well-intentioned. Instead, researchers should develop methodological frameworks that:

- put specific community contexts and priorities at the centre,
- recognise distinct cultural protocols and practises,
- acknowledge the varying historical experiences of colonisation,
- respect different approaches to knowledge transmission,
- honour diverse ways of knowing and being.

This attention to diversity carries significant implications for research practise, including:

- extended timeframes for relationship building and community engagement,
- flexible methodological approaches that may be adapted to local contexts,
- resource allocation that supports meaningful community engagement,
- recognition of the multiple valid ways of conducting and evaluating research.

Implementing these principles will necessitate a fundamental transformation of research practises and training. We must go beyond a superficial acknowledgement of diversity to adopt methodological frameworks that truly respect and respond to the rich complexity of Indigenous peoples, cultures, and knowledge systems.

## 6 Conclusion: employing an Indigenous rights–based approach in settler colonial contexts

Associate Professor Levon Blue, HDR Coordinator/Course Coordinator, Office of the Deputy-Vice-Chancellor (Indigenous Engagement) at the University of Queensland, reflected that she had not previously considered a rights-based approach to education:

I had previously taught preservice education courses where UNDRIP was mentioned but never applied. Peter [Anderson] demonstrated with conviction how … school issues … could be overcome using an Indigenous rights-based approach … [Adopting] an Indigenous rights-based approach to curriculum design and/or embedding Indigenous perspectives ensured that Indigenous perspectives were front and centre … This is an approach that can't be argued against... as it is an inherent right that Indigenous peoples have to education. (November 2024)

The intimate relationship between colonialism and research continues to perpetuate the historical oppression and dispossession of Indigenous peoples. This issue extends beyond academic oversight to encompass systemic practises of cultural violence, knowledge suppression, and institutional discrimination. From anthropological studies that reinforce racist ideologies to non-consensual medical experimentation, research has often acted as a tool of colonialism, contributing to broader patterns of dispossession, cultural erasure, and colonial domination (Tuhiwai Smith, 2021).

Indigenous rights-based research offers a meaningful response to this troubled history. It is not just a theoretical concept; it has the potential to transform contemporary research practises. It extends beyond the superficial acknowledgement of historical injustices and the problematic rhetoric surrounding reconciliation, reconceptualising research relationships, methodologies, and objectives. This will lead to a genuine justice-based reconciliation grounded in the principles of UNDRIP, fostering the “right relationship” (Goerke and Anderson, 2021) between Indigenous and non-Indigenous peoples. By centring Indigenous rights and perspectives, Indigenous rights-based research seeks to change research from a tool of oppression into a means of community empowerment and healing.

This transformation will require a fundamental reconsideration of the relationship between research institutions and Indigenous communities. Acknowledging past harms and actively committing to new forms of engagement that prioritise Indigenous sovereignty and self-determination are critical for addressing historical trauma. Educational researchers who adopt this approach must recognise these historical traumas while working towards healing and transformation, and they must transform their research within the broader context of Indigenous resurgence and decolonisation. This necessitates a deep consideration of existing power dynamics, Indigenous community priorities, and the potential for their research to either promote or hinder community healing and cultural revitalisation. In other words, research must serve as a tool for Indigenous empowerment rather than perpetuate patterns of colonial harm.

As stated by Sato, director of the teacher certification program at International Christian University in Japan, “thinking about Indigenous issues from a rights-based approach …. was particularly meaningful and contributed greatly when I was working on a book project about Indigenous people in Japan” (November 2024).

Indigenous rights-based research is a transformative paradigm that fundamentally reshapes how research is conceptualised, conducted and disseminated in settler colonial contexts. In this paper, we have focused on five key aspects—Indigenous people as data, protocols of engagement, privileging Indigenous knowledge systems, community benefit and doctoral research training.

Indigenous people as data refers to how traditional statistical analyses reinforce the deficit narrative surrounding Indigenous achievement. By employing Indigenous rights–based methodologies such as within-cohort peer-matching analysis, researchers can enhance their understanding of Indigenous educational experiences and outcomes while ensuring methodological rigour.

Protocols for engagement involve fostering meaningful, reciprocal partnerships and dialogue with Indigenous communities. Researchers must dedicate adequate time to develop these relationships, ensure explicit and informed consent processes, and maintain ongoing dialogue throughout the research process.

The privileging of Indigenous knowledge systems within institutional structures and practises necessitates a fundamental shift in the validation, transmission, and application of knowledge in academic contexts. This transformation challenges traditional academic hierarchies and fosters an environment for Indigenous ways of knowing and being.

Community benefit is a crucial measure of research success. To ensure genuine community benefit, research objectives must be redefined to prioritise Indigenous perspectives and needs, ensuring that the outcomes serve the community's interests rather than merely advancing individual academic careers or fulfilling institutional priorities.

Finally, addressing doctoral training emphasises the significance of culturally responsive education that prepares both Indigenous and non-Indigenous PhD candidates with the knowledge and skills necessary to effectively apply Indigenous rights-based approaches.

According to Russ Fox, a lecturer in behavioural sciences in education at Monash University,

… the Indigenous rights–based approach is about heart transformation first. The orientation to research questions, methods, analyses and all the nuts and bolts of the work all flow out of this transformation … It is a joyous and liberating transformation that expands possibilities. (November 2024)

This transformation will necessitate a sustained commitment at both the institutional and individual levels, including:

- a radical reform in how research is conceptualised and conducted,
- the development of institutional structures that support Indigenous protocols,
- the creation of culturally safe spaces for Indigenous researchers,
- the establishment of meaningful, reciprocal community partnerships,
- a guarantee that research will confer direct community benefits.

The challenges of implementing Indigenous rights-based approaches include institutional barriers, a lack of trust, and the complexities of navigating diverse knowledge systems. These obstacles highlight the need for a sustained and collective commitment to embrace the significant structural changes necessary to decolonise research practises, transform institutions, and develop genuine partnerships with Indigenous communities. Current perceptions of research excellence, valid knowledge, and impact assessment must be re-evaluated.

As Indigenous peoples continue to assert their rights to self-determination and sovereignty, research methodologies must be urgently developed to support, rather than obstruct, these efforts. Indigenous rights–based approaches offer a vital framework for accelerating the journey towards decolonised research methodologies. Our collective experience of the five aspects demonstrates that while transformation can be challenging, it is both necessary and achievable. Most importantly, when implemented thoughtfully and systematically, these approaches create opportunities for more ethical, relevant, and impactful research that honours Indigenous knowledge systems and contributes to decolonisation efforts in settler colonial states.

While our collaborative approach to developing an Indigenous rights-based methodology represents an evolving initiative, it marks a critical step in aligning research practises with the broader global decolonial project. The delayed adoption of UNDRIP in settler-colonial states like Australia underscores the deep-seated resistance within institutional power structures that this approach seeks to transform. Yet, the increasing implementation of Indigenous rights-based approaches across academic disciplines signals a meaningful shift that moves beyond symbolic acknowledgements and reconciliation statements towards substantial structural change.

Our work contributes to this essential trajectory by operationalising UNDRIP principles at the epistemic level, demonstrating how Indigenous leadership in research can effectively challenge colonial knowledge systems while creating new pathways for justice and community empowerment. As Indigenous peoples worldwide assert their sovereignty and right to self-determination, approaches like the IRBA provide practical frameworks for ensuring that academic research supports rather than hinders these vital movements towards decolonization and Indigenous resurgence.

## Data Availability

The original contributions presented in the study are included in the article/supplementary material, further inquiries can be directed to the corresponding author.

## Footnotes

^1^In this paper, we use the term ‘Indigenous' when discussing generic issues that are recognised internationally. “Indigenous” is the term used in the UN Declaration of the Rights of Indigenous Peoples (United Nations, 2007). For the presentation of the case study in Australia, we use the term “Aboriginal” being the collective term for one of the two legally recognised Indigenous communities in Australia, those Indigenous people of the Australian mainland and Tasmania. This is in distinction of the people of the Torres Strait Islands who are the other community who are collectively recognised as Indigenous. The Indigenous higher education expert who has led the development of this Indigenous rights-based approach is an Australian Aboriginal person.
